# Evaluating Postoperative Immobilization Following Hip Reconstruction in Children With Cerebral Palsy

**DOI:** 10.7759/cureus.30270

**Published:** 2022-10-13

**Authors:** Sean Tabaie, Kevin Cho, Omar Tarawneh, Alana Sadur, Aribah Shah

**Affiliations:** 1 Orthopaedic Surgery, Children's National Hospital, Washington DC, USA; 2 Orthopaedic Surgery, George Washington University School of Medicine and Health Sciences, Washington DC, USA; 3 Neurological Surgery, New York Medical College, Westchester, USA

**Keywords:** surgery, hip displacement, cerebral palsy, open reduction, abduction pillow, hip spica

## Abstract

Objectives

Currently, there is no standardized protocol for postoperative immobilization techniques in patients with cerebral palsy undergoing hip reconstructive procedures. The purpose of this study was to evaluate the effects of several methods of postoperative immobilization and to determine which postoperative immobilization technique has the fewest complications.

Materials and methods

A retrospective cohort study of pediatric patients with cerebral palsy who underwent hip reconstructive procedures, in which a hip spica cast, Petrie cast, or abduction pillow was placed for postoperative hip immobilization, was conducted. Patients who underwent revision surgery and those without cerebral palsy were excluded from the analysis. The final cohort consisted of 70 cases. Demographics, laterality of surgery, procedure type, hip immobilization technique, and 30-day postoperative complications were recorded. Complications were defined as those related to casting immobilization, such as re-dislocation or loss of surgical fixation, and soft tissue complications, such as pressure ulcers or any superficial or deep wound infection.

Results

Of the 70 patients, 27 received spica casting, 28 received Petrie casting, and 15 received an abduction pillow. The complication rates, as defined in the methods section, were 14.8% for the spica cast group, 17.9% for Petrie cast, and 26.7% for abduction pillow. There was no significant difference in complication rates among spica cast, Petrie cast, or abduction pillow groups (P=0.76).

Conclusions

There was no significant difference in length of stay, pain control duration, or complication rates among the three methods of immobilization. Clinicians should be advised of the comparable outcomes among the postoperative immobilization techniques.

## Introduction

Cerebral palsy (CP) is a heterogeneous group of neuromuscular disorders that manifests as increased muscle tone, joint deformities, and impaired mobility and posture [1]. Among children, CP is the leading cause of disability and the most commonly diagnosed neuromuscular disorder with a prevalence of 2-3 per 1,000 births [2-4].

Hip subluxation, dislocation, and dysplasia of the acetabulum are common sequelae of cerebral palsy (CP), and the risk of displacement is directly associated with the Gross Motor Function Classification System (GMFCS), which is traditionally used to grade patients on functional ability and limitations [5]. In fact, it is estimated that approximately one-third of children with CP will experience these symptoms associated with hip dysplasia during the course of their life [6]. These pathologies cover a broad clinical spectrum resulting from a combination of spastic muscles placing abnormal forces on the hip joint resulting in subluxation, and bony deformities [7,8]. Treatment of these hip disorders includes non-operative management such as physical therapy, bracing/orthotics, medications for spasticity (botulinum toxin, baclofen), and operative management including soft tissue release, selective dorsal rhizotomy, and bony deformity procedures, yet many aspects of the postoperative management are not well described in the literature [9-12].

Femoral, acetabular osteotomies and at times an anterior hip open reduction are the primary surgical interventions for hip disorders in CP patients, but there is a lack of consensus on postoperative immobilization following surgery [13,14]. Cast immobilization, such as hip spica and Petrie casting, can be used for postoperative immobilization to protect the hip reconstruction and prevent early postoperative complications if there is concern for instability; however, it may give rise to complications owing to its highly restrictive nature, such as avascular necrosis of the femoral head, joint stiffness, decubitus ulcers, precluding visual inspection of the surgical site, additional time for application after the surgical procedure, pain, difficulty with cast and individual hygiene, and can be difficult to manage by families [15]. Due to the highly restrictive nature of spica and Petrie casting, additional complications arise which may affect quality of life, such as issues with hygiene, positioning, and transportation. However, the rationale behind using such restrictive methods of immobilization is that it becomes increasingly important to maintain a stricter control on postoperative abduction and immobilization as the surgical burden increases or in the setting of open reductions. Conversely, abduction pillows were thought to be an alternative method of immobilization that are less restrictive, more easily placed and removed, and typically reserved for patients with less surgical burden and less complicated cases, often those not requiring an open reduction. In the setting of early re-dislocation or loss of implant fixation, abduction pillows were either not used or switched to a spica or Petrie cast for additional immobilization. However, in recent years, some surgeons have begun to prefer to utilize the abduction pillow even in open reductions, with the hope that this allows for patients to progress to weight bearing in a stander as soon as tolerated. There is some concern that liberal use of the abduction pillow may result in loss of abduction gained from adductor lengthening, however some centers also report no increase in adverse events attributable to the method of immobilization, such as residual subluxation or early re-dislocation. Despite this, abduction pillows are still not widely used due to the lack of available supporting evidence.

Currently, the method of postoperative immobilization is determined by surgeon preference as there have been no studies comparing the three immobilization techniques. This study sought to evaluate different methods of postoperative immobilization in patients with cerebral palsy following hip reconstruction to determine which method of postoperative immobilization leads to the fewest complications.

## Materials and methods

After obtaining approval from the Children's National Hospital Institutional Review Board (Pro00013335), a retrospective cohort study of patients was performed. Inclusion criteria consisted of patients 18 years or younger who underwent hip reconstruction from January 1, 2009 to December 2, 2019 at Children's National Hospital. We identified 227 patients who underwent unilateral or bilateral proximal femoral osteotomies, pelvic osteotomies, or hip open reductions. It is important to note that none of the patients had concomitant botulinum toxin or chemodenervation injections at the time of their hip reconstruction surgery. Patients were excluded if they underwent revision surgery or were not diagnosed with cerebral palsy. Demographics, laterality of surgery, procedure type, postoperative hip immobilization technique, and 30-day postoperative complications were recorded. Complications were defined as those related to casting immobilization, such as re-dislocation or loss of surgical fixation, and soft tissue complications, such as pressure ulcers or any superficial or deep wound infection.

Statistical analysis

Summary statistics on demographic and baseline patient characteristics were presented as means with standard deviation for continuous data and frequencies with percentages for categorical data. Continuous and categorical baseline variables were compared between three types of immobilization using one-way ANOVA test and Chi-square test or Fisher’s exact test (if expected cell count <5) respectively. Normality assumptions were checked using a statistical test (e.g., Shapiro Wilk test) as well as graphical methods (e.g., histogram, q-q plot). Only length of stay was found to be significantly deviated from normal.

Length of stay was compared using a non-parametric Kruskal-Wallis test. We compared complication rates between three groups using a Chi-square test followed by a multivariable logistic regression analysis adjusted for the aforementioned variables. All statistical tests were two-sided and performed at the 0.05 level of significance. Statistical analysis was performed using R statistical software, version 4.0.0 (R Core Team, 2020).

## Results

There was a total of 70 patients who met our inclusion criteria; 27 (39%) were placed in a spica cast, 28 (40%) in a Petrie casting, and 15 (21%) in an abduction pillow. The demographics and baseline patient characteristics were compared between groups (Table 1). There were significant differences between the groups with respect to age, height, and weight (P=0.002, 0.023, 0.007 respectively). There were no significant differences in patient American Society of Anesthesiologists (ASA) classification (P=0.999), but there were significantly more comorbid conditions in the abduction pillow group.

**Table 1:** Demographic and baseline patient characteristics SD = Standard Deviation, ASA = American Society of Anesthesiologists P-values were obtained from one-way ANOVA test for continuous data, and Chi square/Fisher’s exact test for categorical data

Patient characteristics	Overall (N=70)	Petrie (N=28)	Pillow (N=15)	Spica (N=27)	P value
Age (years), mean (SD)	9.6 (5.7)	12.1 (6.3)	10.1 (3.9)	6.8 (4.8)	0.002
Height (cm), mean (SD)	118.6 (24.5)	125.1 (23.7)	126.1 (19.7)	108.8 (24.6)	0.023
Weight (kg), mean (SD)	26.6 (12.2)	30.4 (13.4)	29.7 (9.7)	20.9 (10.3)	0.007
BMI (kg/m^2^), mean (SD)	17.6 (3.8)	18.3 (4.6)	17.5 (2.8)	16.9 (3.2)	0.371
BMI percentile, mean (SD)	54.6 (37.2)	53.0 (34.8)	56.1 (38.6)	55.2 (39.9)	0.968
ASA, n (%) Class 1 Class 2 Class 3 Class 4	1 (1.6) 9 (14.8) 50 (82.0) 1 (1.6)	0 (0.0) 3 (13.0) 19 (82.6) 1 (4.3)	0 (0.0) 2 (13.3) 13 (86.7) 0 (0.0)	1 (4.3) 4 (17.4) 18 (78.3) 0 (0.0)	0.999
Unilateral, n (%)	56 (80.0)	21 (75.0)	10 (66.7)	25 (92.6)	0.092
Epidural analgesia, n (%)	35 (50.0)	12 (42.9)	9 (60.0)	14 (51.9)	0.546
Procedure, n (%) Combined Femur Hip	44 (62.9) 16 (22.9) 10 (14.3)	20 (71.4) 6 (21.4) 2 (7.1)	5 (33.3) 8 (53.3) 2 (13.3)	19 (70.4) 2 (7.4) 6 (22.2)	0.008
Open Reduction, n (%)	35 (50.0)	15 (53.6)	2 (13.3)	18 (66.7)	0.004
No. of comorbidities, mean (SD)	6.5 (2.7)	6.9 (2.7)	7.5 (2.8)	5.6 (2.4)	0.047

There were significant differences between the groups with regard to the procedures performed. The abduction pillow group had a higher percentage of patients who underwent only femur procedures (53%), while both the spica and Petrie cast groups had more patients who underwent combined procedures (71% and 74%). Finally, there was a difference in the groups with regards to the percentage that underwent an open reduction of the hip, with 13% of patients in the abduction pillow group undergoing that procedure compared to 54% and 67% in the Petrie and spica cast groups respectively.

The complication rates, as defined in the methods section, were 14.8% for the spica cast group, 17.9% for Petrie cast, and 26.7% for abduction pillow (Table 2). A chi-squared test was used to analyze the data, and there was no significant difference in complication rates among spica cast, Petrie cast, or abduction pillow groups (P=0.634). We also compared the length of stay, duration of pain control modality, and complications between the three groups. Multivariate analysis revealed that length of stay and pain control duration were not significantly different among the three groups.

**Table 2:** Variables for each group, including complication rates SD = Standard Deviation,  IQR = Interquartile Range P-values were obtained from one-way ANOVA test or Kruskal–Wallis test (if skewed) for continuous data, and Chi square test for categorical data

Variables	Petrie (N=28)	Pillow (N=15)	Spica (N=27)	P value
Length of Stay (days), median (IQR)	5.1 [3.3, 7.0]	5.2 [3.5, 7.8]	3.8 [3.0, 5.2]	0.042
Foley Duration (days), mean (SD)	2.1 (1.1)	2.6 (1.1)	2.3 (1.4)	0.589
Pain Control Duration (days), mean (SD)	2.5 (1.3)	2.7 (0.7)	2.2 (1.2)	0.465
Complications, n (%)	5 (17.9)	4 (26.7)	4 (14.8)	0.634

To control for patient demographic differences, a multiple linear regression was used. This adjusted analysis did not show any significant differences in the length of stay, and mean pain control durations between the three groups (Table 3). The complication rate was also not found significantly different across the three groups in the multivariable logistic regression analysis (Table 4).

**Table 3:** Multiple linear regression analysis adjusted for age, BMI percentile, procedure type, and immobilization type CI = Confidence Interval

Variables	Pillow - Petrie difference (95% CI)	P value	Spica - Petrie difference (95% CI)	P value
Length of Stay (days)	-0.33 (-4.41-3.76)	0.874	-1.55 (-5.14-2.03)	0.388
Foley Duration (days)	0.37 (-0.61-1.35)	0.452	0.25 (-0.59-1.1)	0.547
Pain Control Duration (days)	0.68 (-0.48-1.84)	0.243	-0.72 (-1.68-0.23)	0.133

**Table 4:** Multivariable logistic regression analysis comparing complication rates adjusted for age, BMI percentile, procedure type, and immobilization type aOR = adjusted Odds Ratio, CI = Confidence Interval

Types of immobilization	aOR (95% CI)	P value
Petrie	ref	ref
Pillow	0.60 (0.08, 4.16)	0.612
Spica	0.58 (0.08, 3.85)	0.569

## Discussion

Currently, there is no consensus on the proper immobilization technique following hip reconstruction surgery in children with CP. Prior work suggests no differences between spica and Petrie casting with regard to complication profiles and similar results have been seen when comparing spica casting to use of an abduction pillow. Abduction pillows are one example of a less constrained positioning equipment that offer several advantages over casting; mainly the ability to assess the skin and care of the child are easier in an abduction pillow. This study sought to compare these immobilization methods to generate data regarding the risks associated with them. Our data suggest that there are no significant differences in complication rates and length of stay between any of the postoperative immobilization techniques. This finding suggests that there are no significant clinical ramifications of using a less restrictive immobilization type (i.e. abduction pillow) for patients undergoing hip surgery, especially those undergoing femoral, acetabular or combined procedures.

Recently, Truong et al. compared spica casting to short-leg Petrie casting in patients with CP following hip reconstruction [16]. They found no difference in hip stability, narcotic pain medication usage, or complication profile. The use of an abduction pillow postoperatively, following hip reconstruction surgery, has been suggested as an alternative method of immobilization. In a single surgeon retrospective review, Albrektson et al. compared 32 patients either placed in an abduction pillow and knee immobilizer, or a spica cast, with a follow-up period of five months [15]. Of the 21 patients in the abduction pillow group, 17 had no complications, and in the spica cast group, 10 of the 11 patients had no complications. The authors noted that one of the complications in the abduction pillow group was not related to the postoperative immobilization method. Due to its restrictive nature, spica casting is also rarely associated with injuries to vessels and nerves [17,18]. They noted that it may be more important to use a spica cast for children with osteopenic bone at risk for implant failure, which is more common in non-ambulatory children. Truong et al. suggested that there was no significant advantage in hip stabilization when comparing use of spica versus Petrie immobilization, but that more children who received spica casting experienced femoral fragility fractures [16,19].

Given the known complications of spica cast treatment, determining the safety profile of less restrictive methods of postoperative immobilization is important [16,20]. Our data suggest no significant differences in complication rates among the three methods of immobilization. Given these data, it seems that abduction pillows are as effective as spica and Petrie casts without the additional risks of cast-related complications. Additionally, spica and Petrie casting are also burdensome on the caretakers, posing challenges with perineal care and hygiene. Given the retrospective and non-randomized nature of our study, a larger prospective study comparing these types of immobilization methods is warranted. Included in this prospective study should be surveys on parent satisfaction and ease of care questionnaires which would greatly bolster the discussion. Given our results, we suspect that casting might be phased out of the postoperative care plan in the right population of hip reconstruction patients with CP.

The results of this study are promising as it describes similar efficacy of pillows as a less restrictive and less burdensome method of postoperative immobilization compared to its more restrictive counterparts. However, the present study has several limitations. This study was a single-center retrospective review, which results in a selection bias due to lack of randomization. The study also had a small sample size, including only two patients who underwent an open anterior hip reduction and were immobilized with an abduction pillow, which could have affected the statistical significance of the analysis. Heterogeneity of the patient population of this study is another limitation. For example, when analyzing the demographic data in Table 1, there was a statistically significant (P=0.002) difference in patient age among the immobilization groups; patients in the spica group had a mean age of 6.8 years, compared to 12.1 and 10.1 in the Petrie groups. Since patients over six years old generally do not tolerate spica casting due to its highly restrictive nature, this immobilization method is often limited to use in younger patients. Another important covariate that was not accounted for in this study was the GMFCS score due to inconsistent records in patient charts. In addition to the GMFCS score, a more detailed reporting of patient comorbidities and surgical burden would also help to better understand the severity of the patient’s condition, as well as the overall medical complexity of the patients in each group. The heterogeneity of the patient population introduces potential confounders that were not able to be accounted for in this study.

Height and weight data were retrieved from patient medical records, from which we calculated BMI and BMI percentile. We observed that this was an inaccurate figure in many patients, given the challenge to measure these parameters in patients with disabilities, who may have trouble standing, for example.

In summary, there was no significant difference in complication rates among the three methods of immobilization. These results suggest that patients with CP undergoing hip reconstruction do not necessarily benefit from spica or Petrie casting in the setting where an open reduction is not performed. Further analysis of patients immobilized with an abduction pillow after skeletal osteotomies in conjunction with an anterior hip open reduction is needed to definitively recommend its use versus a more restrictive option in the setting of an open reduction. Despite the limitations, to the best of our knowledge, this is the first study to document complication rates of postoperative immobilization techniques in children with CP undergoing hip surgery. We are hopeful that this study will be the first step in allowing practitioners to standardize postoperative hip immobilization in this patient population.

## Conclusions

The optimal immobilization method after hip reconstruction in children with CP that requires skeletal osteotomies with or without hip open reduction remains unclear. Our study aimed to evaluate postoperative immobilization methods to determine which form of immobilization produces the fewest complications. We observed no significant differences in complication rates and length of stay across all postoperative immobilization techniques assessed. Our findings provide evidence that there are no significant clinical ramifications of using less restrictive immobilization types such as abduction pillows in patients undergoing hip reconstruction surgery. Further analysis of patients immobilized with an abduction pillow after skeletal osteotomies in conjunction with an anterior hip open reduction is needed to definitively recommend its use versus a more restrictive option in the setting of an open reduction. Therefore, multicenter and prospective trials are warranted to gather sufficient data to standardize postoperative immobilization in children with CP.
