# Correction: Evaluating Postoperative Immobilization Following Hip Reconstruction in Children With Cerebral Palsy

**DOI:** 10.7759/cureus.c80

**Published:** 2022-11-04

**Authors:** Sean Tabaie, Kevin Cho, Omar Tarawneh, Alana Sadur, Aribah Shah

**Affiliations:** 1 Orthopaedic Surgery, Children's National Hospital, Washington DC, USA; 2 Orthopaedic Surgery, George Washington University School of Medicine and Health Sciences, Washington DC, USA; 3 Neurological Surgery, New York Medical College, Westchester, USA

The author list of this article has been corrected at the request of the authors. Second author Kevin Cho and third author Omar Tarawneh were erroneously omitted from the submitted manuscript and this error was not noticed until after publication. As stated by the submitting author, "The reason these two individuals had their names left off is because the draft that was sent to me initially to be uploaded to Cureus did not have the updated title page attached. I therefore did not have the complete author list and I was given the final title page after completing the submission process. I again apologize for this issue."

